# Mutual and asynchronous anticipation and action in sports as globally competitive and locally coordinative dynamics

**DOI:** 10.1038/srep16140

**Published:** 2015-11-05

**Authors:** Keisuke Fujii, Tadao Isaka, Motoki Kouzaki, Yuji Yamamoto

**Affiliations:** 1 Research Center of Health Physical Fitness and Sports, Nagoya University, Furo-cho, Chikusa-ku, Nagoya, 464-0814, Japan; 2 Research Fellow of the Japan Society for the Promotion of Science, Japan; 3 Faculty of Sports and Health Science, Ritsumeikan University, 1-1-1 Noji-higashi, Kusatsu, Shiga 525-8577, Japan; 4 Graduate School of Human and Environmental Studies, Kyoto University, Yoshida-nihonmatsu-cho, Sakyo-ku, Kyoto, 606-8501, Japan; 5 Research Center of Health Physical Fitness and Sports, Nagoya University, Furo-cho, Chikusa-ku, Nagoya, 464-0814, Japan

## Abstract

Humans interact by changing their actions, perceiving other’s actions and executing solutions in conflicting situations. Using oscillator models, nonlinear dynamics have been considered for describing these complex human movements as an emergence of self-organisation. However, these frameworks cannot explain the hierarchical structures of complex behaviours between conflicting inter-agent and adapting intra-agent systems, especially in sport competitions wherein mutually quick decision making and execution are required. Here we adopt a hybrid multiscale approach to model an attack-and-defend game during which both players predict the opponent’s movement and move with a delay. From both simulated and measured data, one synchronous outcome between two-agent (i.e. successful defence) can be described as one attractor. In contrast, the other coordination-breaking outcome (i.e. successful attack) cannot be explained using gradient dynamics because the asymmetric interaction cannot always assume a conserved physical quantity. Instead, we provide the asymmetric and asynchronous hierarchical dynamical models to discuss two-agent competition. Our framework suggests that possessing information about an opponent and oneself in local-coordinative and global-competitive scale enables us to gain a deeper understanding of sports competitions. We anticipate developments in the scientific fields of complex movement adapting to such uncontrolled environments.

Living organisms in social biological systems interact mutually by anticipating other’s actions and executing optimised solutions even in conflicting situations. Computational neuroscience has revealed individual agents’ cognition (e.g. anticipation^1^) and motor control^2^,^3^ through verification that the measured behaviour follows the prediction of the theoretical models. However, competitive mutual anticipation and action in multiagent systems (e.g. each agent perceives relevant information, decides on and then executes suitable actions) exhibit a great diversity in the behavioural forms. Thus, it remains unclear as to how the multiagent system behaviours are derived from redundant inter- and intra-cognitive and motor modules^4^. Nonlinear dynamics have generally been studied to explain such complex phenomenon by using simple mathematical models such as those in electrical circuit simulations^5^ and fluid dynamics^6^. Social behaviour in human movement systems has also been modelled using differential equations and potential functions to describe the interactions among agents of the system^7^. This concept was based on the idea that control of the system is distributed over a multiagent system, which can be represented as an emergence of self-organisation^8^,^9^, rather than being controlled by individual agent’s localised internal structures^10^, e.g. various organs in an organism such as the brain and muscles. In contrast, however, social behaviours in nature are executed by localised internal cognitive (e.g. sensory organ and brain) and motor modules (muscles). These modules can be understood through an internal model for sensorimotor integration^11^. Moreover, in localised internal structures, for example, self-organised intentional brain dynamics^12^ and nervous systems^9^ can be mathematically modelled. These seemingly contradictory viewpoints were derived from different assumptions about the hierarchical system structure; therefore, a comprehensive understanding of the complex human movements at multiple spatiotemporal scales is required. Especially in abrupt changes in human behaviour which results in complexity, researchers have proposed hybrid systems^13^ that consist of discrete inputs as the motor command (i.e. cognitive output) and the continuously dynamical motor system. This switching behaviour has been studied theoretically^13^ and numerically^14^ and observed extensively in swing movements in sports^15^, muscle activities^16^,^17^, walking^18^ and standing control^19^. Methodologically, researchers have focused on either adaptive intra-agent biological movements^7^,^15^,^19^ towards an uncertain external environment or a multi-agent system^20^,^21^,^22^ without external input. However, even these frameworks cannot explain the hierarchical structure of complex behaviour between competitive inter-agent systems and coordinative intra-agent systems. The present study focuses on the hierarchical structure of competitive behaviour consisting of global inter-agent systems and local intra-agent cognitive and motor systems, especially in sport competitions where mutually quick decision making and execution are required.

Two competitive agents should abruptly change the whole two-agent system to achieve their objectives (e.g. escape^23^ or attack^24^) from the deadlock or skirmish situations which have been revealed in tag-play^21^ and martial arts^20^,^22^. Nonlinear dynamics have also investigated bifurcation phenomena defined as discontinuous qualitative changes in a system caused by the continuous change of some control parameter (bifurcation parameters)^25^. Collective behaviours such as in traffic congestion^26^ and animal groups^27^ have been explained by the bifurcation phenomena to model the individual agents’ internal structures. The previous study on competitive movement systems also modelled the behaviours of the two-agents as nonlinear oscillators^20^,^22^ in which the two-agents were assumed to interact symmetrically; however, abrupt system changes should be formulated as an asymmetric interactive model wherein the movement of an agent (e.g. attacker in a ballgame and forward car in traffic) influences the movement of another agent (e.g. defender and backward car), but the latter has a different or no effect on the former. This asymmetric interaction cannot always assume the physical quantities to obey the law of conservation such as the restoring force in previous nonlinear oscillators^7^,^20^. Therefore, the competitive two-agent system should also be modelled considering two asymmetric input–output relations based on the results of the actual measurements of both, the actions of the attacker and that of the defender.

We previously focused on the one-on-one dribble in a ballgame^24^ in which the roles of the attacker and defender were fixed and the outcome was determined as either a successful attack or defence (Fig. 1A). Previous results have demonstrated that a defender’s better preparatory body state before taking the defensive step, which was defined as the maximum value of vertical ground reaction force (*Fz*)^28^, helps in preventing a delay of the defensive step initiation and promotes a successful defence^24^ (Fig. 1C, Video S2,3). These studies suggest that the preparatory body state related to the defender’s delay may be a candidate control parameter of the two-player system in the specific game; however, the general framework to discuss the effect of this delay on the entire two-player system behaviour remains unclear. The interactive behaviour should be revealed by the simulations to manipulate this parameter. The individual visuo-motor delay was derived from both cognitive and motor factors. The cognitive delays were derived by information processing such as anticipation of others’ movements^29^ (e.g. correct anticipation reduces delay). Motor delay was generated before the primary objective action (i.e. before outputting a response) and was previously represented as the preparatory body state by the measurement of the peak in *Fz*^28^ and the joint torque fluctuation^30^. We should therefore clarify the hierarchical structures in competitive two-player systems which have both cognitive and motor systems. The preparatory body state would be seemingly regarded as the localised internal state of an individual motor module, however, we should examine the effect of utilisation of the opponent’s preparatory body state on the outcome by manipulating to switch the observation module (attacker’s cognitive module in Fig. 2). In this case, the preparatory body state should be termed as the localised motor state, rather than the internal state (i.e. internal-and-external state in Fig. 2). We thus sought to determine whether the individual localised motor state generally affects the two-player system behaviour by constructing a minimal competitive attack-and-defend model (Figs 1B, 2 and Video S1), implementing a preparatory body state and comparing the model results with the actual measurement data. We then introduced a stochastic variable as the preparatory body state to simplify our main problem in this study to explain the hierarchical structure of the complex behaviour.

## Results

### Modelling of attack-and-defend system

We first configured the rules of the games by defining an attacker’s win as the outcome where the inter-agent distance in straight lines exceeds a minimum penetration distance (0.5 m, see Methods) within 5 s. The preparatory body states (attacker: PSo and defender: PSx) were implemented as stochastic parameters in uniform-distributed open interval (0, 1), which resulted in the defender’s and attacker’s reaction delay through a Sigmoidal function. Additionally, when the attacker repeats maximal acceleration, penalty delay was added to the attacker’s movement. The attacker’s cognitive model output maximal acceleration if the predicted maximal inter-agent distance was over the minimum penetration distance using PSx information and if not, it output feint acceleration at low speed (details are given in Methods). The defender’s cognitive model simply output the identical motor command as the attacker.

**Figure 1:**
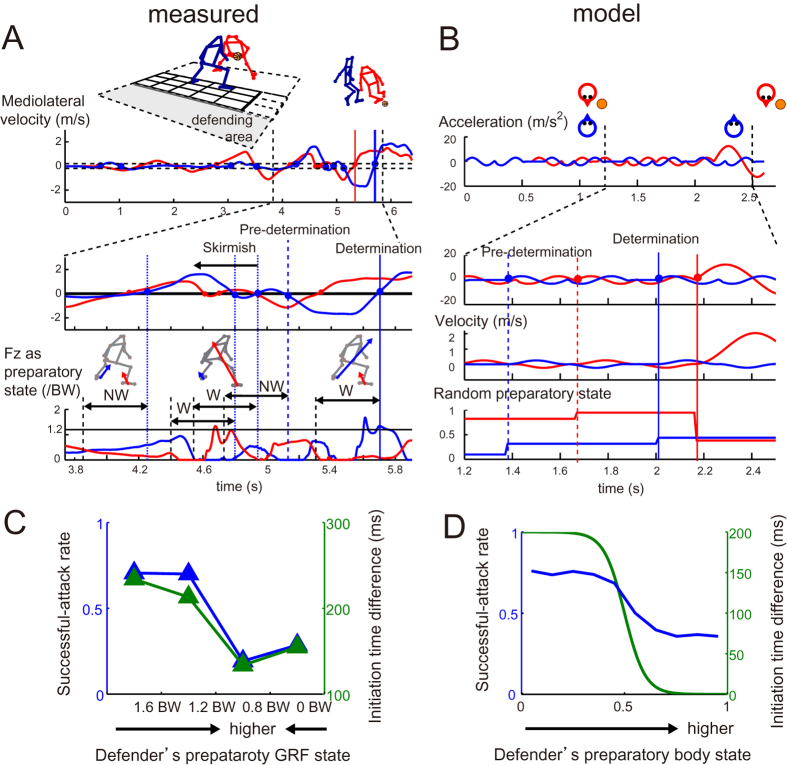
Measurement and simulation results. (**A**) Time series of measured defender’s (blue) and attacker’s (red) mediolateral velocity and vertical ground reaction forces (*Fz*) of defender’s leading foot (blue) and trailing foot (red) in a successful-attack trial. We separated three phases based on the defender’s initiation (blue dot), defined as the initial rise in velocity. The determination phase was defined as the period from 0.4 s before the defender’s initiation to the defender’s initiation in which the outcome of the game was determined (successful attack or failure). The pre-determination phase was the period immediately previous to the determination phase. The skirmish phase was all remaining phases. In each phase, we categorised into two ground reaction force (GRF) states (NW: non-weighted and W: weighted). (**B**) Time series of simulated defender’s (blue) and attacker’s (red) acceleration and velocity. Two types of discrete motor command (maximum and feinting) were input and transformed into sinusoidal acceleration in motor module. (**C**) Measured successful-attack rate histogram as a function of preparatory GRF state. We assumed that the preparatory body state increases with the decreased defender’s preparatory GRF state. (**D**) Simulated successful-attack rate histogram as a function of defender’s preparatory body state in observed (solid) and non-observed (dashed) condition. τ_initial_ and τ_penalty_ were set to 0 and 0.2 s, respectively.

This model was simulated 10,000 times per conditions such as defender’s initial delay, attacker’s penalty delay and non-/observed condition investigating the effect of an attacker’s observation of PSx. Figure 3A–F presents the successful-attack rate as a function of the final PSo and PSx and defender’s initial delay. First, the successful-attack rate in the observed condition was higher than that in the unobserved condition regardless of defender’s initial delay (Fig. 3A–C, main effect: *F*_*1,54*_ = 10.1, *p *= 0.025; there was no significant interaction. Fig. 3D–F had a similar trend). Second, the successful-attack rate was increased with the defender’s initial delay (main effect: both *F*_*2,54*_ = 7.7, *p* = 0.012, *η* = 0.60). Third, in the observed condition, the attacker’s penalty delay had little influence on the system outcome (Fig. 3G–I), which indicates that the attacker precisely predicted a minimum penetration distance (no main effect: *p* > 0.05). In the non-observed condition, there was a significant main effect of penalty and initial delay (penalty: *F*_*4,135*_ = 6.9, *p* = 4.0 × 10^−5^, *η*^2^ = 0.36, initial delay: *F*_*4,135*_ = 22.7, *p* = 3.1 × 10^−9^, *η*^2^ = 0.59, no significant interaction) but no significant main effects in one-way ANOVA (all *p* < 0.05). We set the defender’s initial and attacker’s penalty delay to 0 and 0.2 s as fixed values in the system, to accommodate the widest possible range of successful-attack rate (Fig. 3A) and qualitatively to achieve the effect of attacker’s penalty delay on the successful-attack rate in non-observed condition (Fig. 3G–I), respectively. It was noteworthy that the final PSx greatly affected the successful-attack rate as well as the actual data (Fig. 1C,D), whereas the effect of the PSo on the system behaviour were smaller than that of the PSx. We then investigated whether the defender’s and attacker’s reaction delay coefficients influenced the difference in the change in successful-attack rate (Fig. 4). When the defender’s delay coefficient was the same or less than the attacker’s, the successful-attack rate was nearly zero or defender almost always won, whereas no defender’s delay had the opposite effect. Along with the measured win-and-lose game, we configured the delay coefficients to 0.1 s for the attacker and 0.2 s for the defender. In this case, the successful-attack rate range as a function of defender’s preparatory state in the observed condition was larger than that as a function of the attacker’s (0.45 vs. 0.08), probably because the effect of the attacker’s observation of PSx was added to the difference in the delay coefficients.

### Dynamical system behaviour in the model and measurements

For the trajectory of the two-player system, the phase portraits of the inter-agent distance in the model and the measured data (Fig. 5A,B) were similar in that the original point was considered to be the attractor, and the system diverged from it when the attacker moved at high speed. The trajectories in the successful-defence trials returned to the attractor, whereas those in the successful-attack trials further diverged (simulation was ended when the outcome was determined). The difference in relative velocity and acceleration amplitude between the two outcomes in measured data (Fig. 5C,D) was probably derived from the attacker’s prediction to avoid bumping into the defender after the attacker’s prediction to fail in attack^24^, rather than from competition dynamics of the one attractor and divergence from the attractor. Specifically in determination phase to determine the outcome (see Methods and Fig. 1C,D) of modelling and measurement, although the distributions of velocity and acceleration difference were similar in both outcome (Fig. 5E,G,I,K), the distribution of the peak value was shifted to high value during the successful-attack compared to the successful-defence (Fig. 5J,H,L, all *p* < 10^−5^). The histograms of the order parameters in all three phases were presented in Fig. S1 and S2. The parameters in pre-determination and skirmish phase were similar in successful attack and defence trials for both whole and peak histograms.

### Localised motor state transition analysis

For the investigation of the discrete localised motor state transition in the whole game (Fig. 4), the state was divided into high and low preparatory state based on the half value (PSx = 0.5), which corresponds to non-weighted and weighted states in the measurement, respectively. The model replicated the actual state transition in the determination phase. In the actual data in the non-weighted state, they defended successfully in 79% of the trials (observed model: 60%; unobserved model: 68%), whereas in the weighted state this percentage dropped to 30% (observed model: 25%; unobserved model: 37%). This tendency was largest in the actual, followed by the observed and unobserved models in order (*χ*^2^ (1) = 22.0 and *V* = 0.46, *χ*^2^ (1) = 1201.3 and *V* = 0.35, and *χ*^2^ (1) = 869.4 and *V* = 0.29; all *ps* < 10^−6^). With respect to the transition before the determination phase, although the probabilities were homogeneous in both modelling and measurement (all *Vs* < 0.09), number of the two states were more biased in the measurements than the modelled (measured: all *Vs* > 0.29; model: all *Vs* < 0.20). For the measurement data, there was greater number of the non-weighted state in the skirmish phase and the pre-determined phase (38 and 48 trial, compared with 12 and 26 trial in the weighted state, respectively), which changed to the weighted states in the determination phase.

## Discussion

In the present study, we investigated sports movement dynamics in asymmetrical attack-and-defend competitions. The phase portraits revealed that one synchronous outcome (i.e. successful-defence) and the other two-agent coordination-breaking outcome (i.e. successful-attack) can be described as one attractor and divergence from the attractor, respectively. Specifically, in the determination phase of modelling and measurement, the distributions of the peak value in velocity and acceleration differences were shifted to a high value during the successful-attack compared with that for the successful-defence (Fig. 5J,H,L), suggesting that the an attack-and-defend system behaviour changed abruptly rather than smoothly throughout the determination phase. In previous studies focused on the dynamical system of complex biological movements, researchers modelled relatively stable intra-^15^ or inter-personal^20^,^22^ coordination using a nonlinear oscillator, in which the state of the system changed smoothly during the analysed phases. To take advantage of the opponent, however, this two-agent coordination needs to be broken^24^; therefore, the presence of a restoring force in the oscillator cannot be assumed. Consequently, on the global scale, this abrupt two-agent coordination-breaking cannot always assume the conserved quantity and thus cannot be explained entirely in terms of gradient dynamics (i.e. potential function). Instead, the movement delay derived from the hierarchical dynamical model consisting of discrete cognitive and continuous motor modules and asynchronous inter-agent mutual anticipation and action can explain both the outcomes in the two-player system. From this hard-coded model viewpoint, the abrupt two-agent coordination-breaking phenomenon can be explained from the attacker’s discrete decision on and execution of the successful attack, which breaks the assumption of the previous human movement model (i.e. nonlinear oscillator). From the local internal structure^9^,^12^ to the global inter-agent coordination^7^, many complex human systems can be explained in terms of self-organisation; however, our results suggest that this globally competitive and locally coordinative movement should be considered at multiple spatiotemporal scales, rather than in terms of mono-scale self-organisation. It should be noted that this phenomenon cannot be regarded as a phase transition or bifurcation, because these explanations need a priori assumption of a detail-free model, as is common with various phenomena. Using measurement and modelling in the present study, two players move asynchronously, especially in successful-attack trials (Figs 1C,D and 5J,H,L). This asynchronous interaction exhibits a great diversity of forms in cellular automata^31^ as in the modelling and actual behaviour of a swarm of soldier crabs^32^. Our results also provide a framework to discuss the diverse asynchronous two-agent competitive mutual anticipation and action system using the minimal model. The framework is suggested as a first step towards understanding the emergence of complex biological behaviour and can be applied in interdisciplinary research domains such as neurophysiology^2^, biomechanics^19^, psychology^33^ and game theory^23^. The paradigms of neurophysiology and biomechanics mainly focused on physical machineries of individual motor systems; in contrast, the paradigms of psychology and game theory mainly focused on navigation mechanisms to elucidate the rules by which agents make decisions^34^. Our globally competitive and locally adaptive model was considered as a minimal interdisciplinary model outside these frameworks because our model includes minimal physical machineries and navigation mechanisms. However, a more precise implementation of both cognitive and motor modules is needed for gaining further understanding of the phenomena as discussed below. Practically, we can anticipate development in control of both one’s own body and the uncontrolled environments, e.g. controlling robots in a complex task^35^, impairment and recovery of motor functions in a broad sense such as in falling^36^ or sports^24^ and in improvement of skills in competitive sports such as ballgames^24^ and martial arts^20^,^22^.

Our results reveal that the preparatory body state might be a candidate control parameter in the virtual attack-and-defend dynamical system behaviour. In the case of the win-and-lose situation where the results of the modelling and measurement outcomes on players at an intermediate level were similar, the defender’s preparatory state might be one candidate control parameter in the two-player system. In the remaining cases, the attacker almost always won or lost depending on the delay coefficients. Similar outcomes can be expected from real ballgame situations such as novice versus expert and expert versus expert. We therefore suggest that the preparatory body state as the localised motor state could be a candidate control parameter to explain the qualitative changes in the two-agent system simulation. Especially in the case of the win-and-lose situation, the results suggest that the movement delay derived from the defender’s preparatory state^28^ and the attacker’s computational decision making^29^ while observing the defender’s state and movement (the defender was also modelled to perfectly predict the attacker’s movement) would occur in the real one-on-one dribble games in the determination phase. Before the determination phase, however, the numbers of the two states in the measurements were more biased than those in the model. The transition from the non-weighted to the weighted states may be caused by the control factor which is not included in the model, e.g. the defender’s biomechanical factor. For further understanding of this attack-and-defend competition, the preparatory state itself should be biomechanically modelled, experimentally manipulated and tested as a control parameter of the system. In the measurement, the defender’s *Fz* prior to initiation indicating the preparatory state, however, may be a sport- or expertise-specific indicator. The present preparatory state assumed the ‘mobility’ of the body^37^; thus, it may be expressed as the difference between strategically (or predictively) and biomechanically desired movements. The implementation of biomechanical configurations into the model will produce new principles of adaptive movement mechanics in conflicting and uncontrolled environments.

Our analysis of a real competitive behaviour can be considered as a stochastic hybrid dynamical model which investigates the control of quantitative noisy or stochastic continuous behaviour which can be abstracted in discrete modes in uncertain environments^38^. The present study would provide a minimal model of delayed stochastic hybrid dynamical systems, implementing the localised motor states for the investigation of asymmetric competition in realistic systems such as control for vehicles^39^ based on a mathematical reach–avoid game^38^ or complex inter-agent interaction such as in chase-and-escape problem^23^ including sports^40^. In real biological systems, biological membranes^41^ and biochemical systems^42^ have been modelled by stochastic hybrid dynamical models. Hence, we anticipate further advancements in interdisciplinary biological system modelling by considering individual mechanical body states.

Since time immemorial, humans have always competed against each other. In the The Art of War, Sun Tzu states the following: ‘If you know the enemy and know yourself, you need not fear the result of a hundred battles’^43^. The framework of this study suggests that the possessing information about an opponent and oneself at the globally adaptive and locally competitive scales will facilitate a deeper understanding of competitions, rather than the possessing information only about an individual agent or a two-agent system. For determining the optimal strategy of agents, defenders need to maintain a high preparatory state or decrease the delay. Although the preparatory state was a stochastic parameter in this model, maintaining a high preparatory state can be practically controlled by the defender’s skill in the present study. However, attackers’ have various options such as enhancing their preparatory state, observing the defender’s state and decreasing the attacker’s penalty delay. Furthermore, real attackers use deceptive movements^33^ and adapt to their own or their opponent’s movement characteristics; therefore, further investigations should focus on implementing such higher cognitive functions.

## Methods

### Game structure of the model

In the attack-and defend model, we defined the attacker and the defender moves in straight lines (Supplementary Video 1) because mediolateral movement would be crucial for the determination of successful-attack or successful-defence in the actual measurement^24^. The initial states of the both player’s movement were resting states (inter-agent distance and both player’s velocity were zero). In most actual cases, defenders reacted to the attacker’s movement after visuo-motor delay^44^, we thus designed the virtual defender to react the virtual attacker after the visuo-motor delay, which was defined as the sum of defender’s initial delay τ_1_ and reaction delay as described below. We simulated the model with changing the defender’s initial delay τ_1_ from 0 s to 0.2 s and investigate the effect on the simulation outcome (parameter values were listed at the table in Text S1).

Attacker must actually move longer distance than the defender. In attack-and-defend model, we defined successful-attack as the outcome where the inter-agent distance exceeds the minimum successful-attack distance defined as 0.5 m or the approximately shoulder width within 5 sec. If the successful-attack failed, the outcome was defined as successful-defence. We designed the model to win-and-lose along with results of the actual measurement in order to investigate their determination factor; however, if the two players have the same ability to move, the outcome will be inevitably 100% successful-attack or 100% successful-defence. We thus added the preparatory body state to the motor model in the attack-and-defend model.

**Motor model.** As the simplest motor model, the two kinds of discrete motor command of the attacker and the defender were transformed into continuous sinusoidal acceleration. One was maximal acceleration to penetrate and guard for the attacker and defender, respectively, which was defined as a sinusoidal acceleration whose cycle duration was 0.5 s and whose amplitude was 4 π m/s^2^ to satisfy the minimum successful-attack distance per cycle. Another was feinting acceleration, which was defined as a sinusoidal acceleration whose cycle duration was 0.25 (one action included 2 cycles which were opposite in sign each other) and whose amplitude was π m/s^2^ to assume that attacker accelerates with one leg and decelerates with another leg (i.e. the player eventually returns the same position).

**Figure 2:**
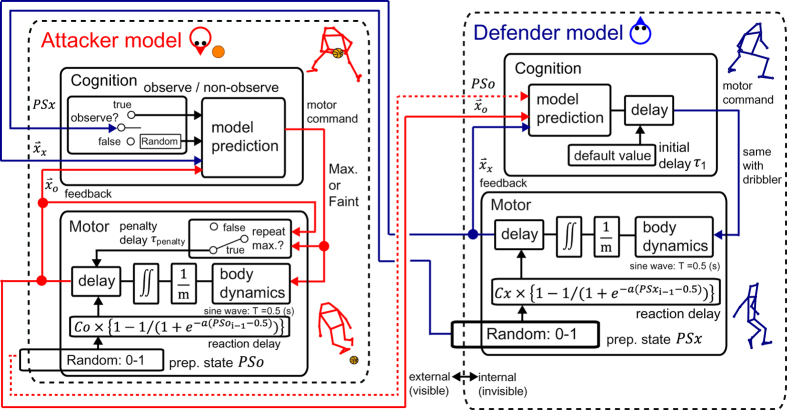
Attack-and-defend model diagram. The preparatory body states (attacker: PSo and defender: PSx) were implemented as random parameters in uniform-distributed open interval (0, 1), which resulted in the defender’s and attacker’s reaction delays (Fig. 1D) through Sigmoidal function (parameter values were shown in Table S1). The attacker’s cognitive model output maximal motor command if the predicted maximal predicted inter-agent distance was over the minimum penetration distance and if not, it output feint motor command at a low speed (detailed model predictions were given in Methods and Text S1). These motor commands were converted into the acceleration through body dynamics (from command into torque) and inertia inversion (from torque into acceleration). In the main text, we skipped this and explained that the cognitive model output the acceleration. The position was then calculated by the second order time integral of the acceleration, accompanied by temporal delays. The defender’s cognitive model simply output the completely same motor command as the attacker. In addition to the stochastic preparatory body state, we examined three parameters in simulations in this study: (1) the defender’s initial delay (τ_1_), (2) attacker’s and defender’s coefficients of delay (Co and Cx, respectively), and (3) attacker’s penalty delay (τ_penalty_) which added to the attacker’s movement when the attacker repeats maximal acceleration. All parameter values were listed in Table S1.

As explained above, we implemented preparatory body state into the attacker’s and defender’s motor model to make the win-and-lose situation. The preparatory body state is the concept expressing ability to execute movement and should be described by biomechanical motor system; however, we simply defined the preparatory state as random parameter in uniform-distributed open interval (0, 1) (0: maximum decrease, 1: no decrease). In attacker’s and defender’s motor model, attacker’s and defender’s preparatory body state PSo_i_ and PSx_i_ were randomly determined during ith movement through movement duration 0.5 s, respectively. Reaction delay was defined by:


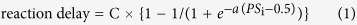


where C was attacker’s and defender’s delay coefficient (expressed as Co and Cx in Fig. 2, respectively), PSi was PSoi and PSxi for the attacker’s and defender’s reaction delay, respectively. A coefficient of the Sigmoidal function ‘a’ was set to 20. The attacker’s and defender’s delay coefficients was validated in the simulation results section.

Additionally, when the attacker repeats maximal acceleration, attacker’s penalty delay τ_penalty_ was added to the attacker’s movement, which assumes difficulty in motor control or fatigue along with the actual measurement. If the directly-previous movement did not have the maximal acceleration, τ_penalty_ was zero. We simulated the model with changing τ_penalty_ from 0 to 0.3 s and we investigated the effect on the simulation outcome Therefore, total reaction delay τ_i_ was obtained by:


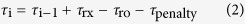


where i was a natural number which means ith action and τ_ro_ and τ_rx_ were the attacker’s and defender’s reaction delay, respectively.

### Cognitive model

The attacker’s and defender’s cognitive models input oneself and opponent information and discrete motor command above. The attacker’s cognitive model input its own and defender’s position, velocity and preparatory body state and output motor command based on the criteria: (1) if the model judged the next movement will achieve successful-attack, the model output maximal acceleration in the direction based on the model prediction. In the model prediction, to calculate maximal predicted inter-agent distance (PIAD) to win the game for the attacker, the model must predicted delay τ_pi_:


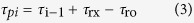


where i was a natural number except that τ_p1_ was zero. The model can then calculate maximal PIAD by obtaining the maximum value in the PIAD as follows:


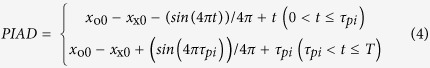


where x_o0_ and x_x0_ were attacker’s and defender’s position when the ith movement starts respectively. Details are given in Text S1. (2) If the model judged the next movement will not achieve successful-attack, the model output feint acceleration in the opposite direction of the difference in both player’s velocity when the attacker’s movement begins. We also investigated the effect of attacker’s observation of PSx on the simulation outcome by setting conditions: observed condition and non-observed condition where attacker inputs different random parameter in uniform-distributed open interval (0, 1) as predicted PSx. The defender’s cognitive model simply input the attacker’s movement information and output the completely same motor command as the attacker because we assumed the defender moves after the attacker and then there was no opportunity to utilise other prediction cue such as the attacker’s preparatory state or defender’s own movement information.

### Simulation

The above model was simulated at 10,000 times per conditions such as defender’s initial delay, attacker’s penalty delay and observed or non-observed condition. The numerical integration was executed by the 4th order Runge-Kutta method. We evaluated the outcome of the attack-and-defend model on a successful-attack rate. We also analysed the preparatory state transition probabilities as the whole-time one-on-one game competitive dynamics^24^. Phase division was based on the actual measurement described below.

### Measurement in actual one-on-one dribble

Measurement data was completely the same as our previous study^24^ and described in Supplementary Text S2 in detail.

### Statistical Analyses

**Figure 3:**
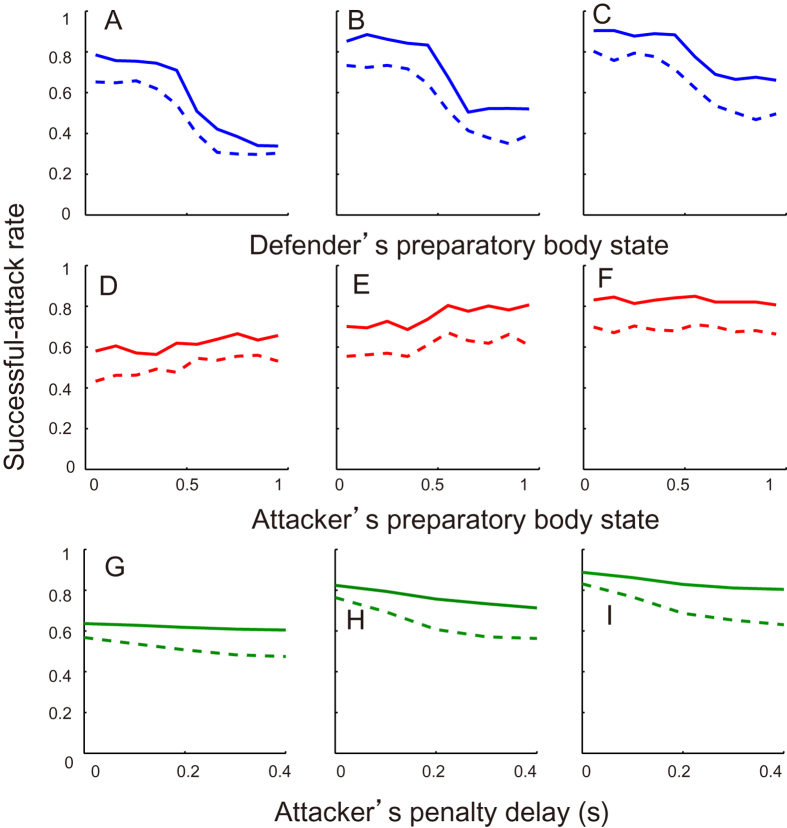
Effect of parameters on the attack-and-defend system. Simulated successful-attack rate histogram as a function of defender’s (**A–C**) and attacker’s preparatory body state (**D–F**) and attacker’s penalty delay (**G–I**) in observed (solid) and non-observed (dashed) condition. Columns of A, B and C indicated that defender’s initial delays τ_initial_ were set to 0, 0.1, and 0.2 s, respectively. All data in Fig. 3A-F and Fig. 3G–I was submitted as Supplementary Dataset 1 and 2.

**Figure 4:**
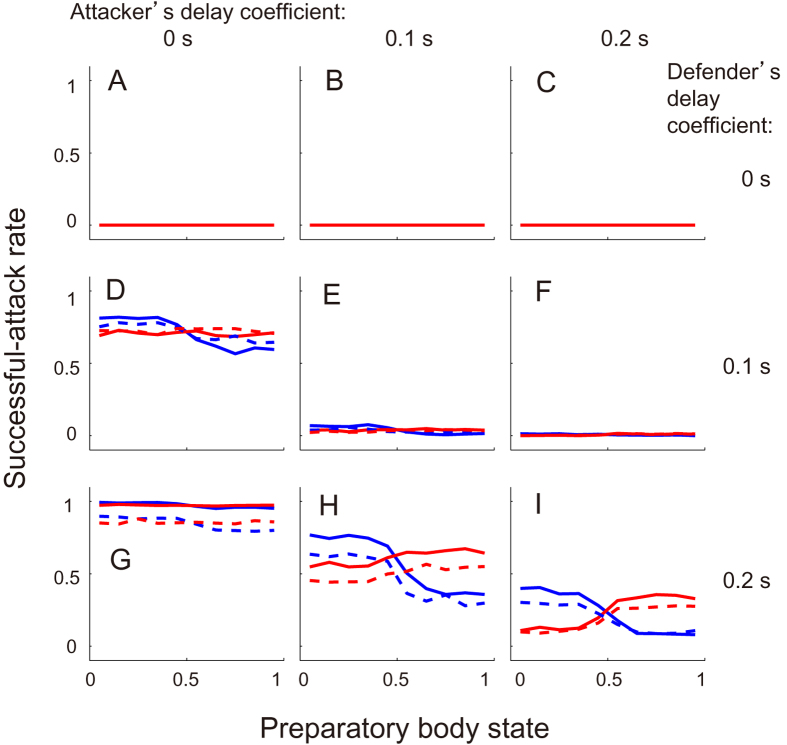
Effect of delay coefficients on the one-on-one system. Simulated successful-attack rate histogram as a function of defender’s (blue) and attacker’s (red) preparatory body state in observed (solid) and non-observed (dashed) condition. Lines of A, D and G were defender’s delay coefficients 0, 0.1 and 0.2 and columns of A, B and C mean attacker’s delay coefficients 0, 0.1, 0.2 s, respectively. τ_initial_ and τ_penalty_ were fixed at 0 and 0.2 s, respectively.

**Figure 5:**
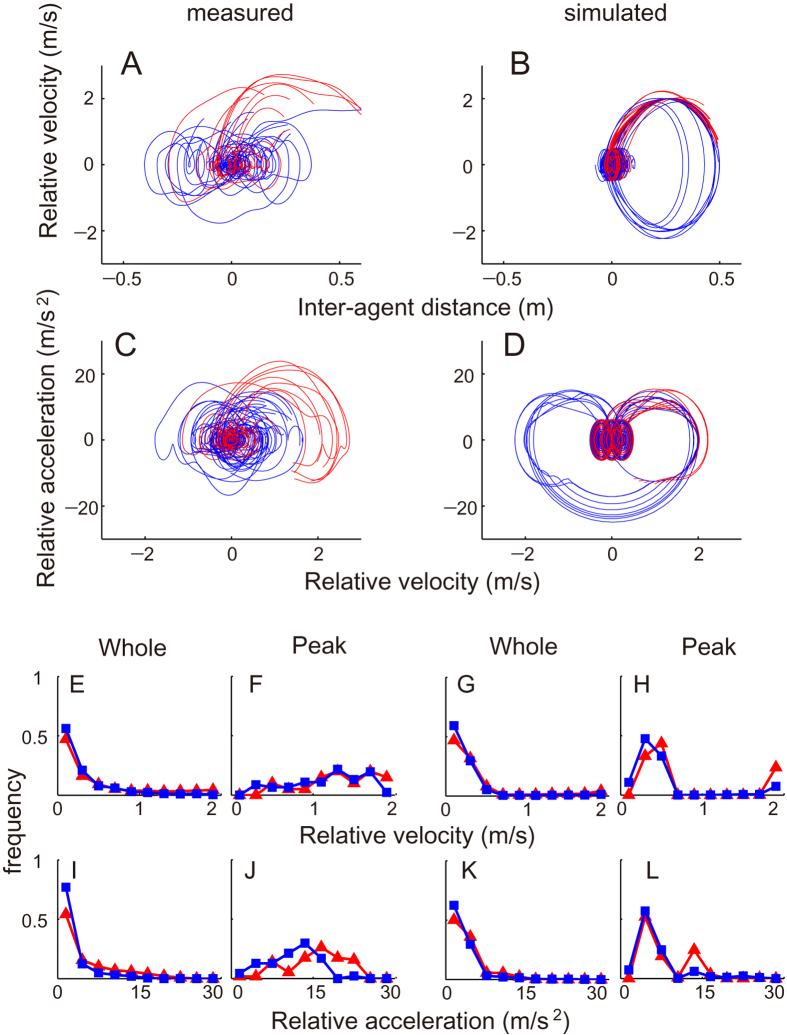
Phase portrait and histogram of order parameters in two-player system. Trajectory in the phase plane of interplayer distance (**A,B**) and velocity difference (**C,D**) in measured (**A,C**) and simulated (**B,D**) successful-attack (red) and successful-defence (blue) trials. In detail, normalised frequencies of velocity (**E–H**) and acceleration (**I–L**) difference in the determination phase in measured (**E,F,I,J**) and simulated (**G,H,K,L**) successful-attack (red) and successful-defence (blue) trials were presented. All data in Fig. 4E–L was submitted as Supplementary Dataset 3–6.

**Figure 6:**
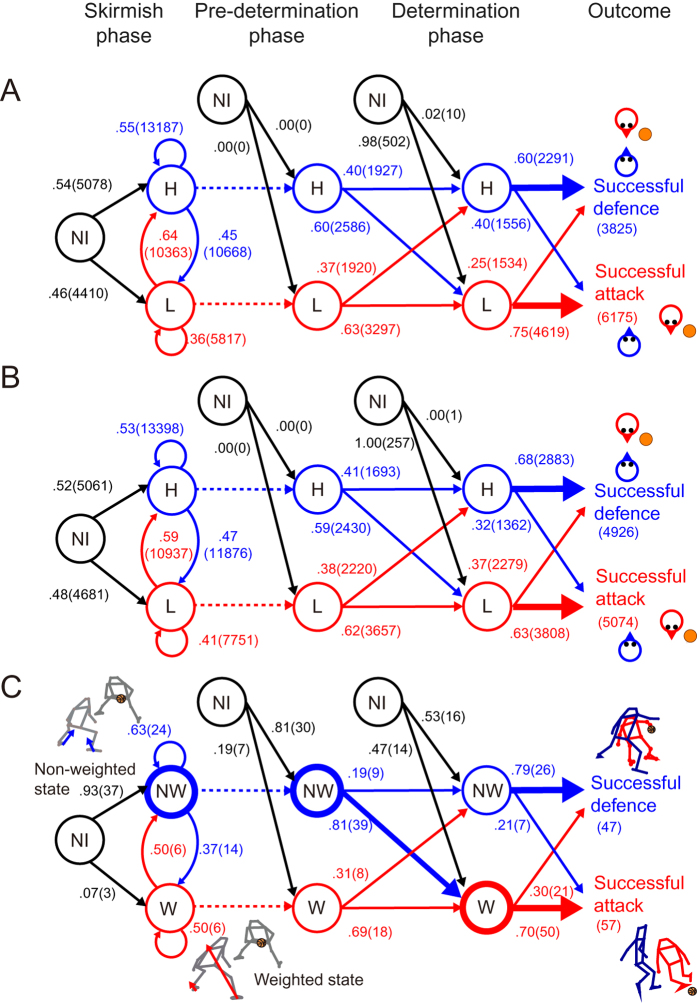
State transition diagrams with the probabilities of the preparatory body state. We confirmed the state transition probabilities in observed model (**A**) non-observed model (**B**) and actually measured data (**C**). The states were categorised into the high (H) and low (L) preparatory state and imaginary no-initiation state (NI) in the model, and into the non-weighted (NW), weighted (W) and imaginary no-initiation state (NI) in measured data. The thickness of arrows represents higher probabilities of state transition, and thickness of circles indicates larger numbers of trials. State transition probabilities (decimal) and numbers (with bracket) were fully described in this figure.

To assess the independent and combined effects of the successful-attack rate (10 bins of the preparatory states), we conducted two-way ANOVAs with τ_1_ and the two observation conditions or with τ_1_ and τ_penalty_. In the latter case (Fig. 3G–I), because the difference between the observation conditions was obvious after the former ANOVA (Fig. 3A–F), we eliminated this factor. Homogeneity of variances was verified except for the ANOVAs of the attacker’s preparatory state probably because of the ceiling effect (Fig. 3D-F). We then performed statistical analyses only using the defender’s preparatory state (Fig. 3A–C), whereas there was a similar trend between the attacker’s and defender’s preparatory state. For comparison of the peak velocity and acceleration between the successful-attack and successful-defence trials, we used Mann–Whitney U-test because of non-normal distribution (Fig. 5F,H,J,L). Chi-squared (χ2) test was performed to measure the relationship among various GRF state transitions in the three phases (Fig. 6). The effect size was estimated using Cramér’s V for Chi-squared test^45^ and used as comparison criteria, because greater number of trials especially in simulation easily resulted in *p* < 0.05. All numerical calculations including these statistical analyses were performed using the MATLAB 2011a Statistical Toolbox (The MathWorks, Inc., MA, USA).

## Additional Information

**How to cite this article**: Fujii, K. *et al.* Mutual and asynchronous anticipation and action in sports as globally competitive and locally coordinative dynamics. *Sci. Rep.* **5**, 16140; doi: 10.1038/srep16140 (2015).

## Supplementary Material

Supplementary Materials

Supplementary Dataset 1

Supplementary Dataset 2

Supplementary Dataset 3

Supplementary Dataset 4

Supplementary Dataset 5

Supplementary Dataset 6

Supplementary Movies S1

Supplementary Movies S2

Supplementary Movies S3

## References

[b1] Berry M. J., Brivanlou I. H., Jordan T. A. & Meister M. Anticipation of moving stimuli by the retina. Nature 398, 334–338 (1999). 10192333 10.1038/18678

[b2] Shidara M., Kawano K., Gomi H. & Kawato M. Inverse-dynamics model eye-movement control by purkinje-cells in the cerebellum. Nature 365, 50–52, 10.1038/365050a0 (1993). 8361536

[b3] Kording K. P. & Wolpert D. M. Bayesian integration in sensorimotor learning. Nature 427, 244–247, 10.1038/nature02169 (2004). 14724638

[b4] Bernstein N. A. Dexterity and Its Development. (Erlbaum, 1996).

[b5] Ueda Y., Abraham R. H. & Stewart B. H. The Road to Chaos. (Aerial Press 1992).

[b6] Lorenz E. N. Deterministic nonperiodic flow. Journal of the Atmospheric Sciences 20, 130–141, 10.1175/1520-0469(1963)020<0130:dnf>2.0.co;2 (1963).

[b7] Haken H., Kelso J. A. S. & Bunz H. A Theoretical-model of phase-transitions in human hand movements. Biological Cybernetics 51, 347–356, 10.1007/bf00336922 (1985). 3978150

[b8] Nicolis G. & Prigogine I. Self-organization in nonequilibrium systems: From dissipative structures to order through fluctuations. (Wiley 1977).

[b9] Kelso J. Dynamic patterns: The self-organization of brain and behavior. (MIT Press, 1995).

[b10] Gibson J. J. An ecological approach to visual perception. (Houghton-Mifflin, 1979).

[b11] Wolpert D. M., Ghahramani Z. & Jordan M. I. An internal model for sensorimotor integration. Science 269, 1880–1882 (1995). 7569931 10.1126/science.7569931

[b12] Kozma R. & Freeman W. J. The KIV model of intentional dynamics and decision making. Neural Networks 22, 277–285, 10.1016/j.neunet.2009.03.019 (2009). 19395236

[b13] Gohara K. & Okuyama A. Dynamical systems excited by temporal inputs: Fractal transition between excited attractors. Fractals-Complex Geometry Patterns and Scaling in Nature and Society 7, 205–220, 10.1142/s0218348x99000220 (1999).

[b14] Gohara K. & Okuyama A. Fractal transition: Hierarchical structure and noise effect. Fractals-Complex Geometry Patterns and Scaling in Nature and Society 7, 313–326, 10.1142/s0218348x99000311 (1999).

[b15] Yamamoto Y. & Gohara K. Continuous hitting movements modeled from the perspective of dynamical systems with temporal input. Human Movement Science 19, 341–371, 10.1016/s0167-9457(00)00018-x (2000).

[b16] Tamaki H. *et al.* Alternate activity in the synergistic muscles during prolonged low-level contractions. Journal of Applied Physiology 84, 1943–1951 (1998). 9609788 10.1152/jappl.1998.84.6.1943

[b17] Kouzaki M., Shinohara M., Masani K., Kanehisa H. & Fukunaga T. Alternate muscle activity observed between knee extensor synergists during low-level sustained contractions. Journal of Applied Physiology 93, 675–684, 10.1152/japplphysiol.00764.2001 (2002). 12133879

[b18] Fu C., Suzuki Y., Kiyono K., Morasso P. & Nomura T. An intermittent control model of flexible human gait using a stable manifold of saddle-type unstable limit cycle dynamics. Journal of the Royal Society Interface 11, 10.1098/rsif.2014.0958 (2014). PMC4223921 25339687

[b19] Bottaro A., Yasutake Y., Nomura T., Casadio M. & Morasso P. Bounded stability of the quiet standing posture: An intermittent control model. Human Movement Science 27, 473–495, 10.1016/j.humov.2007.11.005 (2008). 18342382

[b20] Yamamoto Y. *et al.* Joint Action Syntax in Japanese Martial Arts. Plos One 8, 10.1371/journal.pone.0072436 (2013). PMC3762806 24023740

[b21] Kijima A. *et al.* Switching Dynamics in an Interpersonal Competition Brings about “Deadlock” Synchronization of Players. Plos One 7, 10.1371/journal.pone.0047911 (2012). PMC3489899 23144834

[b22] Okumura M. *et al.* A Critical Interpersonal Distance Switches between Two Coordination Modes in Kendo Matches. Plos One 7, 10.1371/journal.pone.0051877 (2012). PMC3527480 23284799

[b23] Isaacs R. Differential Games: A Mathematical Theory with Applications to Warfare and Pursuit, Control and Optimization. (John Wiley and Sons, Inc., 1965).

[b24] Fujii K., Yoshioka S., Isaka T. & Kouzaki M. The preparatory state of ground reaction forces in defending against a dribbler in a basketball 1-on-1 dribble subphase. Sports Biomechanics 14, 1–17, 10.1080/14763141.2015.1026931 (2015). 25895702

[b25] Badii R. & Politi A. Complexity: Hierarchical Structures and Scaling in Physics. Cambridge University Press (1997).

[b26] Bando M., Hasebe K., Nakayama A., Shibata A. & Sugiyama Y. Dynamical model of traffic congestion and numerical-simulation. Physical Review E 51, 1035–1042, 10.1103/PhysRevE.51.1035 (1995). 9962746

[b27] Couzin I. D., Krause J., Franks N. R. & Levin S. A. Effective leadership and decision-making in animal groups on the move. Nature 433, 513–516, 10.1038/nature03236 (2005). 15690039

[b28] Fujii K., Yoshioka S., Isaka T. & Kouzaki M. Unweighted state as a sidestep preparation improve the initiation and reaching performance for basketball players. Journal of Electromyography and Kinesiology 23, 1467–1473, 10.1016/j.jelekin.2013.08.001 (2013). 24060389

[b29] Fujii K., Shinya M., Yamashita D., Kouzaki M. & Oda S. Anticipation by basketball defenders: An explanation based on the three-dimensional inverted pendulum model. European Journal of Sport Science 14, 538–546, 10.1080/17461391.2013.876104 (2014). 24397711

[b30] Fujii K., Yamashita D., Kimura T., Isaka T. & Kouzaki M. Preparatory body state before reacting to an opponent: Short-term joint torque fluctuation in real-time competitive sports. PLOS ONE 10, 10.1371/journal.pone.0128571 (2015). PMC4449124 26024485

[b31] Ingerson T. E. & Buvel R. L. Structure in asynchronous cellular automata. Physica D 10, 59–68, 10.1016/0167-2789(84)90249-5 (1984).

[b32] Murakami H. *et al.* Emergent Runaway into an Avoidance Area in a Swarm of Soldier Crabs. Plos One 9, 10.1371/journal.pone.0097870 (2014). PMC4026533 24839970

[b33] Brault S., Bideau B., Kulpa R. & Craig C. M. Detecting Deception in Movement: The Case of the Side-Step in Rugby. Plos One 7, 10.1371/journal.pone.0037494 (2012). PMC3372470 22701569

[b34] Nathan R. *et al.* A movement ecology paradigm for unifying organismal movement research. Proceedings of the National Academy of Sciences of the United States of America 105, 19052–19059, 10.1073/pnas.0800375105 (2008). 19060196 PMC2614714

[b35] Yamashita Y. & Tani J. Emergence of Functional Hierarchy in a Multiple Timescale Neural Network Model: A Humanoid Robot Experiment. Plos Computational Biology 4, 10.1371/journal.pcbi.1000220 (2008). PMC2570613 18989398

[b36] Zelik K. E. & Kuo A. D. Mechanical Work as an Indirect Measure of Subjective Costs Influencing Human Movement. Plos One 7, 10, 10.1371/journal.pone.0031143 (2012). PMC3286468 22383998

[b37] Yoshihara Y., Tomita N., Makino Y. & Yano M. Autonomous Control of Reaching Movement by ‘Mobility Measure’. International Journal of Robotics and Mechatronics 19, 448–458 (2007).

[b38] Summers S. & Lygeros J. Verification of discrete time stochastic hybrid systems A stochastic reach-avoid decision problem. Automatica 46, 1951–1961, 10.1016/j.automatica.2010.08.006 (2010).

[b39] Gillula J. H., Hoffmann G. M., Huang H., Vitus M. P. & Tomlin C. J. Applications of hybrid reachability analysis to robotic aerial vehicles. International Journal of Robotics Research 30, 335–354, 10.1177/0278364910387173 (2011).

[b40] Breakwell J. V. & Merz A. W. Football as a differential game. Journal of Guidance Control and Dynamics 15, 1292–1294, 10.2514/3.20985 (1992).

[b41] Buckwar E. & Riedler M. G. An exact stochastic hybrid model of excitable membranes including spatio-temporal evolution. Journal of Mathematical Biology 63, 1051–1093, 10.1007/s00285-010-0395-z (2011). 21243359

[b42] Caravagna G., d’Onofrio A., Antoniotti M. & Mauti G. Stochastic Hybrid Automata with delayed transitions to model biochemical systems with delays. Information and Computation 236, 19–34, 10.1016/j.ic.2014.01.010 (2014).

[b43] Sun T. The Art of War. Oxford Univ. Press. (1963).

[b44] Fujii K., Yamashita D., Yoshioka S., Isaka T. & Kouzaki M. Strategies for defending a dribbler: categorisation of three defensive patterns in 1-on-1 basketball. Sports Biomechanics 13, 204–214, 10.1080/14763141.2014.953983 (2014). 25203390

[b45] Cramér H. Mathematical Methods of Statistics. (Princeton University Press, 1999).

