# Supplementary material for: Mutual and asynchronous anticipation and action in sports as globally competitive and locally coordinative dynamics
Source: Sci Rep. 2015 Nov 5;5:16140. doi: 10.1038/srep16140 (PMC4633604; doi:10.1038/srep16140)
Supplement: Supplementary Materials [file srep16140-s1.pdf]

Supplementary Materials

Mutual and asynchronous anticipation and action in sports as globally competitive and locally coordinative dynamics

Keisuke Fujii, Tadao Isaka, Motoki Kouzaki and Yuji Yamamoto.

Text S1 and S2

Reference (47-49)

Table S1

Figure S1 and S2

Supplementary Dataset 1 to 6 (caption only)

Movies S1 to S3 (caption only)

Text S1. Predicted inter-agent distance. We assumed the point of time at which the attacker started to move as t = 0 and T as the attacker's movement cycle duration. If x_{o0} was the attacker's initial position when $0 \le t \le T$, the predicted attacker position (PAP) was given as follows:

$$\mathrm{PAP} = \iint_0^{\mathrm{t}} \mathrm{Asin}\left(\frac{2\pi \mathrm{t}}{\mathrm{T}}\right) dt^2$$
$$= x_{\mathrm{o0}} + \mathrm{AT}^2(-\sin(2\pi\mathrm{t}/\mathrm{T}))/4\pi^2 + \mathrm{ATt}/2\pi$$

where A was the maximal acceleration amplitude. Note that the attacker's initial velocity was zero.

$$\mathrm{PAP} = x_{\mathrm{o0}} + (-\sin(\pi\mathrm{t}))/4\pi + \mathrm{t}$$

The defender did not move until the predicted delay τ_{pi} (calculated using equation (3) in the main text) or predicted defender's movement initiation at x_{x0}, which was the defender's final position in the previous action or initial position in the current action, i.e. predicted defender position (PDP) was zero when $0 \le t \le \tau_{pi}$. When $\tau_{pi} \le t$, PDP was calculated as follows.

$$\mathrm{PDP} = \iint_0^{\mathrm{t}-\tau_{pi}} \mathrm{Asin}(2\pi\mathrm{t}/\mathrm{T}) dt^2$$

$$= x_{\mathrm{x0}} + \mathrm{AT}^2(-\sin(2\pi\mathrm{t}/\mathrm{T}) + \sin(-2\pi\tau_{pi}/\mathrm{T}))/4\pi^2 + \mathrm{AT}(\mathrm{t} - \tau_{pi})/2\pi$$

Predicted inter-agent distance (PIAD) was calculated by subtracting PDP from PAP.

$$\mathrm{PIAD} = \mathrm{PAP} - \mathrm{PDP}$$

$$= \begin{cases} x_{\mathrm{o0}} - x_{\mathrm{x0}} - \mathrm{AT}^2(\sin(2\pi\mathrm{t}/\mathrm{T}))/4\pi^2 + \mathrm{ATt}/2\pi & (0 < t \le \tau_{pi}) \\ x_{\mathrm{o0}} - x_{\mathrm{x0}} + \mathrm{AT}^2(\sin(2\pi\tau_{pi}/\mathrm{T}))/4\pi^2 + \mathrm{AT}\tau_{pi}/2\pi & (\tau_{pi} < t \le T) \end{cases}$$

In this study, the prediction assumed that the attacker executes the maximal acceleration (cycle duration T was 0.5 s and amplitude A was 4π m/s^2), so PIAD was finally calculated as follows.

$$\mathrm{PIAD} = \begin{cases} x_{\mathrm{o0}} - x_{\mathrm{x0}} - (\sin(4\pi\mathrm{t}))/4\pi + \mathrm{t} & (0 < t \le \tau_{pi}) \\ x_{\mathrm{o0}} - x_{\mathrm{x0}} + (\sin(4\pi\tau_{pi}))/4\pi + \tau_{pi} & (\tau_{pi} < t \le T) \end{cases}$$

Text S1. Supplementary methods of actual measurement.

Participants in actual one-on-one dribble. Measurement data was completely the same as our previous study[21]. In this study, 10 skilled males of a university basketball team (age = 19.2 ± 0.4 years, experience = 8.0 ± 1.8 years [mean ± SD]) participated. The participants provided their written informed consent to participate in this study. The experimental procedures were conducted in accordance with the Declaration of Helsinki and approved by the Local Ethics Committee in the Graduate School of Human and Environmental Studies, Kyoto University (approval number 26-H-12).

Protocol. An attacker with a basketball and a defender were instructed to play a real-time, one-on-one game within a 2.4 × 3.6 m area (mediolateral × anteroposterior space; see Fig. 1A). To obtain the defender's GRFs, the attacker started to move while holding the ball. According to the rule in basketball, the attacker was allowed to move while pivoting (movement while keeping grounded on either foot) and dribbling. The objective of the attacker was to get past the defender and to invade the defended area behind the defender. The experimental task began with the attacker's preferred timing after the experimenter's signal. As in a basketball game, the attacker was not permitted to go across the sideline. The defender aimed to stop the attacker according to the rules of basketball, which allow the defender to stop the attacker from a head-on position only[15]. Each pair performed 12 trials without exhibiting fatigue.

Motion capture. For the kinematics, three-dimensional coordinates of the landmark points were acquired using a 3D optical motion capture system with 16 cameras at 200 Hz (Raptor-EDigital Real Time System, Motion Analysis Corporation, Santa Rosa, CA, USA). Eighteen reflective markers were placed on each participant's body (right and left side of their heads, shoulders, elbows, wrists, hips, knees, ankles, heels and toes). All raw coordinate data points were smoothed using a fourth-order Butterworth low-pass digital filter (8-12 Hz) using residual analysis[47]. The torso displacement that was calculated based on an estimation of body segment parameters[48] were linearly

interpolated from 200 Hz to 1000 Hz because we need high spatiotemporal resolution to evaluate peak GRFs based on the body movement event (i.e., initiation time) described below. To measure the defenders' GRF, 15 force platforms (all 60 × 40 cm) were used with sampling frequency of 1000 Hz (Fig. 1A, TF-4060-B, Tec Gihan, Japan). We detected left or right foot contact separately both by our customised program using the location of the plates and foot markers (ankles, heels and toes) and by visual detection.

Selection and categorisation into successful attack and defense trials.

Defending against an attacker means that the defender is able to deprive the attacker of his or her free movement. The one-on-one subphase ended only after the attacker invaded the defended area (defined as successful-attack trials), crossed the sideline, stopped dribbling (held the ball) or was deprived of the ball by the defender for any reason, e.g. the attacker's poor ball handling. Along with the previous study[15], one of the authors, a domestically certified basketball coach (with 5 years of as a coach and 16 years as a player), visually judged the successful-attack and successful-defense trials based on the above criteria. The computational criterion was not used because the reflective markers were occasionally invisible at the end of the trials as the participants moved out of range of the cameras, particularly in the successful-attack trials in the anteroposterior direction, and due to contact with each other in the successful-defense trials. The total number of one-on-one games (trials) in the present study was 120, of which 57 successful-attack trials and 48 successful-defense trials were successfully captured. In the remaining 15 trials, the recordings of the kinematics or GRFs failed.

Three temporal phases and GRF state transitions. To investigate the GRF state transitions (Fig. 4), we defined three temporal phases by back calculation from the outcome (Fig. 1B) based on the attacker's and defender's initiation which defined as the time to the rising of each mediolateral torso velocity in an absolute coordinate system exceeding 10% of the peak velocity. (1) The determination phase was defined as the period of 400 ms (the duration was explained below) before the defender's initiation

time in which determined the outcome of the one-on-one subphase. (2) The pre-determination phase was defined as the same period before the defender's initiation time immediately previous to the determination phase. (3) The skirmish phase was all remaining phases of the same period other than the determination and pre-determination phases. To establish the beginning of one-on-one subphases, we assume an imaginary non-initiation state to define a first-initiation trial (Fig. 2), in which there was no prior initiation of attacker and defender, to differentiate from the trials that transitioned from a GRF state described below. In these analyses, the trials in which only either the defender or attacker initiated (neither player's peak velocity reached 0.2 m/s) were excluded (Fig. 1B). According to the previous study, the existence or non-existence of both players' initiation was assumed to have no relationship with subsequent results[21].

Preparatory GRF state. We then categorised the preparatory GRF state of the defender before the defender's step initiation in the successful-attack and successful-defense trials into non-weighted and weighted states. The non-weighted state was defined as the state wherein Fz of both of the defender's feet were less than 120% BW, for a period of 400 ms before the defender's initiation time. The other was defined as the weighted state. In the present study, we defined the force threshold (120% BW) based on the value within the slow step initiation of our previous study[22]. We tested other force thresholds (80% and 160% BW) in Fig. 1C and justified the validity of the force threshold in terms of a well-explained outcome-GRF relationship in the three phases. The analysis interval (400 ms) should be adequate considering that the defender takes two or three actions (e.g. moving left, right and then left again) because the defender's movement initiation time also had great variability relative to that of the attacker's, compared with the visual stimulus[22,49].

References

47 **Winter, D. A. *Biomechanics and Motor Control of Human Movement Second edition.*, (A Wiley-Interscience Publication).**

48 **Ae, M., Tang, H. & Yokoi, T. Estimation of inertia properties of the body segments in Japanese athletes. *Biomechanism* 11, 23–33 (1992).**

49 **Fujii, K., Shinya, M., Yamashita, D., Oda, S. & Kouzaki, M. Superior reaction to changing directions for skilled basketball defenders but not linked with specialised anticipation. *European Journal of Sport Science* 14, 209-216, doi:10.1080/17461391.2013.780098 (2014).**

Table S1. Parameter values in simulation model. Diagram in simulation is shown in Fig. 2 of main text. We examined four parameters in simulations in this study.

Parameter	Value in simulation
Initial states of both players	Position: 0 m and velocity: 0 m/s
Amplitude of maximal acceleration	4π m/s^2
Duration of maximal acceleration	0.5 s
Amplitude of feinting acceleration	π m/s^2
Duration of feinting acceleration	0.25 s (2 cycles opposite in sign)
Minimum penetration distance	0.5 m
Preparatory body state (Attacker: PSo, Defender: PSx)	Stochastic variable in uniform-distributed open interval (0,1)—examined
Coefficient of Sigmoidal function (a)	20
Delay coefficients of attacker and defender	0, 0.1 and 0.2 s—examined
Initial delay of defender (τ_1)	0, 0.1 and 0.2 s—examined
penalty delay of attacker ($\tau_{penalty}$)	0, 0.1, 0.2 and 0.3s—examined

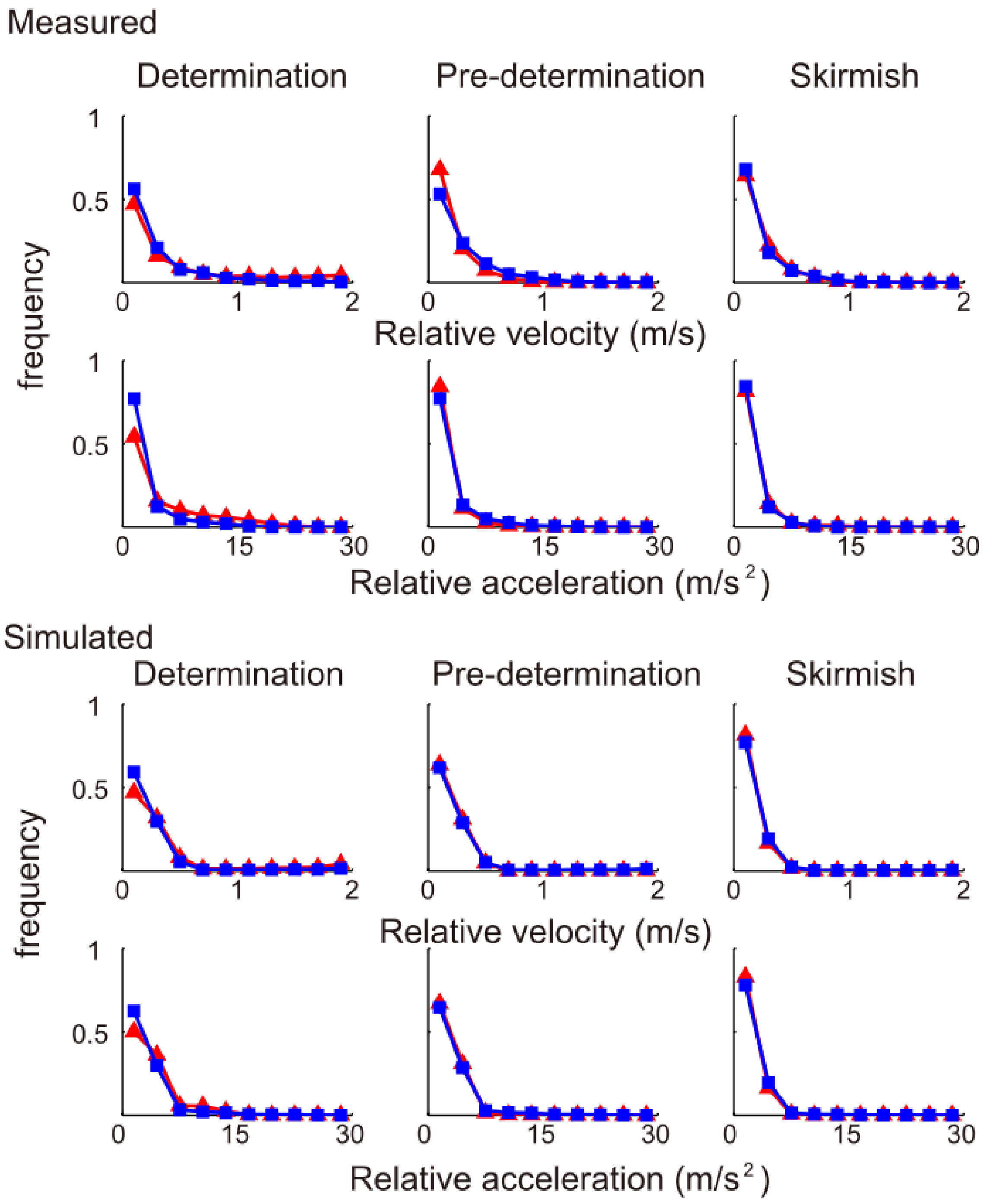

Figure S1. Histogram of order parameters in measured two-player system. Normalized frequencies of velocity and acceleration difference in three phases in measured successful-attack (red) and successful-defense (blue) trials were presented.

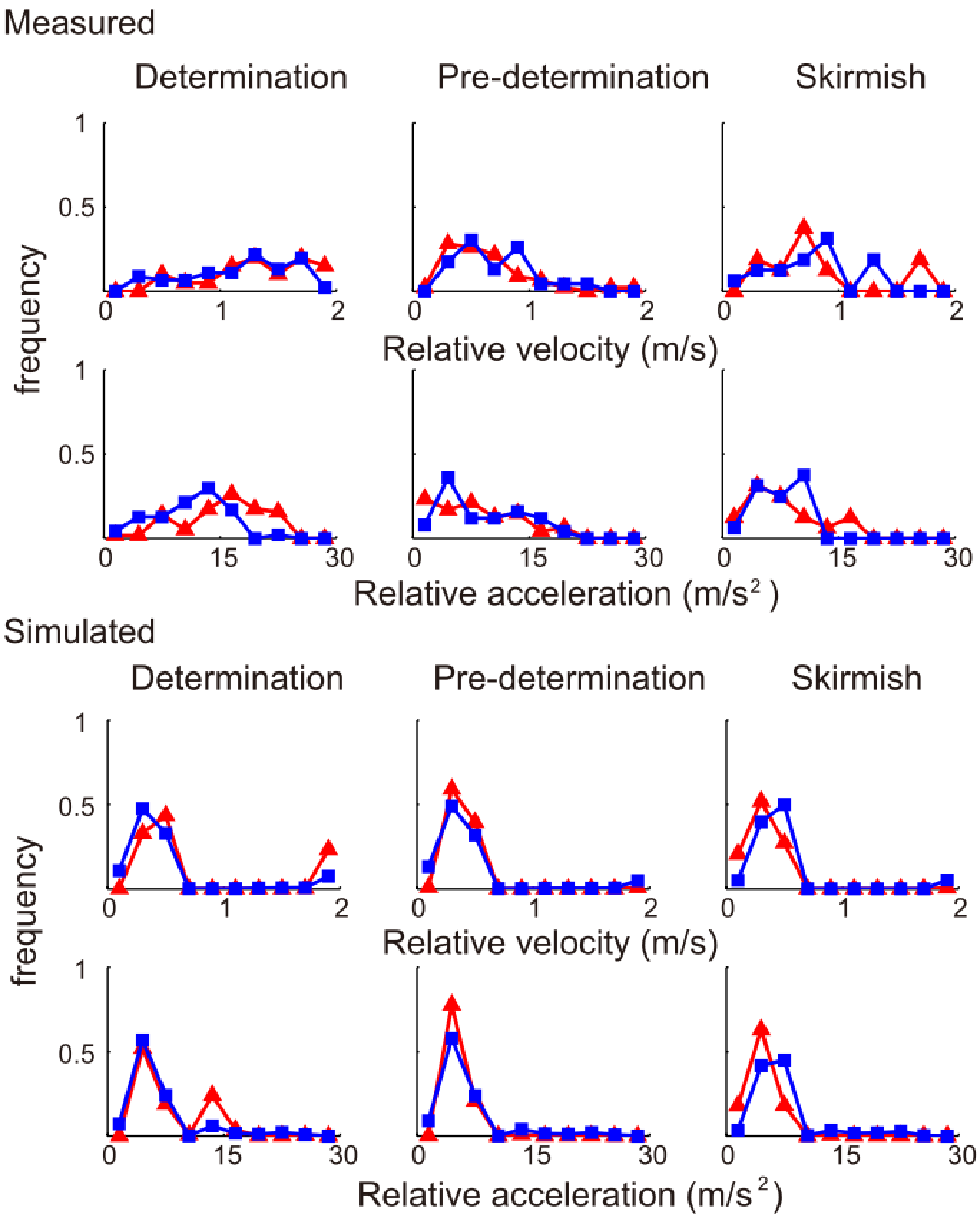

Figure S2. Histogram of order parameters in simulated two-player system. Normalized frequencies of velocity and acceleration difference in three phases in simulated successful-attack (red) and successful-defense (blue) trials were presented.

Supplementary Dataset 1. Simulated 1-on-1 outcome and preparatory body state. Effect of parameters on the attack-and-defend system (Fig. 3 A-F).Simulated 1-on-1 outcome and defender's and attacker's preparatory body state in observed and non-observed condition.

Supplementary Dataset 2. Simulated 1-on-1 outcome and attacker's penalty delay. Effect of parameters on the attack-and-defend system (Fig. 3 G-I). Simulated 1-on-1 outcome and attacker's penalty delay in observed and non-observed condition.

Supplementary Dataset 3. Simulated velocity difference. Velocity difference in the determination phase in simulated successful-attack and successful-defence trials (Fig. 5 G-H).

Supplementary Dataset 4. Simulated acceleration difference. Acceleration difference in the determination phase in simulated successful-attack and successful-defence trials (Fig. 5 K-L).

Supplementary Dataset 5. Measured velocity difference. Velocity difference in the determination phase in measured successful-attack and successful-defence trials (Fig. 5 I-J).

Supplementary Dataset 6. Measured acceleration difference. Acceleration difference in the determination phase in measured successful-attack and successful-defence trials (Fig. 5 I-J).

Movies S1. Video clip showing an example of the simulation of the attack-and-defend model in successful-attack trial. (AVI)

Movies S2. Video clip showing an example of the actual measurement of the non-weighted state in a successful-defense trial. (AVI)

Movies S3. Video clip showing an example of the actual measurement of the weighted state in a successful-attack trial. (AVI)
